# The influence of risk perception in mountaineering on re-participation intentions: a moderated mediation model

**DOI:** 10.3389/fpsyg.2026.1743777

**Published:** 2026-01-16

**Authors:** Huihui Gu, Qi Zhao, Ye Liu, Yutong Yin, Shengchao Bai

**Affiliations:** 1 School of Sports, Leisure and Tourism, Beijing Sport University, Beijing, China; 2 China Winter Sports College, Beijing Sport University, Beijing, China; 3 College of Physical Education and Sports, Beijing Normal University, Beijing, China; 4 Sports Department of Nanjing University of Science and Technology, Nanjing, China

**Keywords:** mountaineering, outdoor recreation, re-participation intention, risk perception, smooth experience, social support

## Abstract

**Objective:**

Mountaineering is a high-risk outdoor activity requiring strong psychological regulation and risk assessment abilities. The study aims to investigate the relationship between risk perception in mountaineering and re-participation intention, specifically focusing on the mediating role of smooth experience and the moderating effect of social support.

**Methods:**

Drawing on Risk Perception Theory and Flow Theory, we proposed a moderated mediation model and surveyed 516 recreational mountaineering participants. Descriptive statistics, reliability and validity tests, and moderated mediation analyses were conducted using SPSS 27.0 (PROCESS 4.1, 5,000 bootstrap resamples).

**Results:**

(1) Risk perception negatively predicted both re-participation intention and smooth experience. (2) Smooth experience positively predicted re-participation intention and mediated the association between risk perception and re-participation intention. (3) Social support moderated the smooth experience and re-participation intention link, such that the positive effect of smooth experience was stronger at higher levels of social support.

**Conclusion:**

Smooth experience constitutes a key psychological pathway through which risk perception relates to re-participation intention, and social support strengthens the behavioral benefits of smooth experience. These findings highlight the joint role of psychological experience and social resources in promoting sustained engagement in mountaineering.

## 1 Introduction

Mountaineering, as a significant form of outdoor recreation, involves activities conducted in natural environments with inherent risks, focused on ascending peaks and traversing alpine terrain (Jackman et al., 2023). Compared to traditional sports, it is more dependent on natural conditions and exhibits greater contextual complexity. While providing both physical and mental enjoyment, mountaineering simultaneously exposes participants to heightened risks, uncertainties, and environmental adaptation pressures. Although mountaineering is gaining widespread popularity, challenges related to risk control and management systems persist. As outdoor sports become more mainstream, risk perception often emerges as a direct barrier to public participation in mountain activities. Individual risk perception acts as a critical link between external risk factors and personal behavioral decisions, directly influencing participants’ willingness to engage in outdoor sports, which in turn significantly affects the long-term, healthy development of the industry (Kim and Jeong, 2024). Importantly, the risks encountered in mountaineering are multidimensional rather than unitary in nature. Beyond general “danger,” mountaineering typically involves (1) environmental risks (e.g., sudden weather changes, altitude, terrain instability), (2) physical injury and physiological risks (e.g., fatigue and altitude-related stress), (3) equipment-related risks (e.g., gear malfunction or inadequate preparation), (4) organizational/management risks linked to route planning, information provision, and emergency support. These risk facets have been repeatedly noted in mountain and adventure contexts and are often experienced in combination, forming a compound risk structure that differs from the more standardized risk types in many traditional sports. This multidimensional risk structure is theoretically relevant because different risk facets may disrupt different conditions for smooth experience, such as perceived control, attention focus, and the balance between skill and challenge, which are prerequisites for entering an optimal experiential state (Šimleša et al., 2018).

In adventurous outdoor activities, risk is unavoidable (Bentley et al., 2007). By exploring the key role and formation mechanisms of risk perception, we can better understand and manage risks, ultimately providing targeted support. Scholars have shown that climbers’ experiential states vary due to differences in environmental conditions, participation motivations, and individual personalities, all of which influence their re-participation intentions (Voorhis et al., 2023). For example, extreme high-altitude climbing can heighten anxiety and trigger altitude sickness, with climbers’ psychological and behavioral responses differing based on personality traits and motivations (Zeng et al., 2024). Furthermore, Flow Theory offers an important theoretical lens for understanding the relationship between risk perception, smooth experience, and re-participation intention (Csikszentmihalyi, 1990). When risk perceived shifts attention toward threat monitoring and reduces perceived control, participants may find it more difficult to maintain deep task absorption and to enter a smooth state in demanding outdoor settings. Previous research has demonstrated that participation behaviors in outdoor adventure activities are strongly shaped by individuals’ risk perception, which influences decision-making processes, emotional responses, and continued engagement (Gilbertson and Ewert, 2015; Vande Vliet and Inglés, 2021). In addition, positive experiential states such as smooth experience have been shown to enhance intrinsic motivation and promote sustained participation in physically challenging activities (Jackson et al., 2023). Furthermore, social factors, including peer support and group norms, play an important role in regulating behavior in high-risk outdoor settings by providing emotional reassurance, informational guidance, and shared behavioral expectations (Xu, 2025). In this context, participation behavior encompasses both intentions and actual actions within a specific activity, shaped by levels of focus, emotional engagement, and the combined influence of internal psychological states and external environmental factors.

Grounded in Risk Perception Theory and Flow Theory, the present study makes three key contributions. First, it extends the literature by contextualizing risk perception within non-professional mountaineering and conceptualizing it as a multidimensional construct, thereby addressing a gap in context-specific evidence within high-risk outdoor sports. Second, it elucidates the mediating role of smooth experience in the relationship between risk perception and re-participation intention, revealing the experiential mechanism through which perceived risk influences behavioral decisions. Third, by introducing social support as a moderator, it highlights how social resources interact with psychological experience to strengthen the conversion of positive states into behavioral intentions, offering practical insights for fostering sustainable participation in outdoor sports.

## 2 Literature review and hypotheses establishment

### 2.1 Risk perception and re-participation intentions

Risk Perception Theory, originating from psychology, pertains to an individual’s subjective perception and understanding of various objective hazards in the external environment (Li et al., 2023). This theory emphasizes that intuitive judgments and subjective feelings play a central role in shaping individual cognition. Scholars have categorized risk perception into different dimensions based on their research fields and subjects. Priest and Baille (1987) define risk perception as the cognitive process in which individuals subjectively evaluate actual risks in exploratory environments. Sitkin and Weingart (1995) define risk perception as an individual’s assessment of the level of risk in a given situation. In the sports domain, risk perception is often categorized into physical injury risks, environmental risks, facility risks, technical risks, and management risks (Shiro and Eiji, 2021; Haegeli and Pröbstl-Haider, 2016). Risk perception can be understood as an individual’s subjective judgment of potential threats, losses, or uncertainties encountered during activity participation (Grima et al., 2021). The degree and intensity of risk perception are influenced by individuals’ cognitive appraisals, experience levels, and activity-specific characteristics. Different types of participants may perceive the same situation differently. For instance, recreational mountaineers in group tours tend to avoid locations with high perceived risks, while adventure enthusiasts may adopt a more positive attitude towards risk, seeking moderately higher risks to enhance the excitement of the experience. In sports tourism research, although risk is often considered a potential motivator for adventure, risk perception may exert positive effects by eliciting emotional arousal in adventure tourism contexts, without directly influencing behavioral intentions (Li et al., 2015). However, some scholars have attempted to demonstrate that latent and actual risks in adventure sports may inhibit participation. From the perspective of Signaling Theory, the public expression of concern over risk in national policy documents and outdoor sports media is a way of signaling the market, indicating high awareness of uncertainty and danger, as well as a focus on risk prevention (Teng et al., 2025). In high-risk activities such as mountaineering, risk perception is usually negatively correlated with participation willingness, affecting individual satisfaction, loyalty, and behavioral intentions (Zhang et al., 2015).

Re-participation intention reflects participants loyalty to an activity. Research on re-participation in the tourism and marketing fields is well-established (Wilopo and Inggang, 2024), but research in sports, particularly on outdoor mountaineering, is limited. In this study, re-participation intention is defined as the intention to engage in similar mountaineering activities in the future, which is an effective means of revitalizing outdoor mountaineering sports. Research shows that when the abilities of mountain sports participants align with the demands of the environment, their recognition of the environment is stronger, allowing them to focus better on the current activity and increasing their intention to participate (Karakullukcu et al., 2025). Prospect Theory and Behavioral Decision Theory suggest that individuals tend to exhibit risk aversion when facing uncertainty and potential losses, often opting for more conservative strategies (Kahneman et al., 1982; Kahneman and Tversky, 1979). For example, mountaineers tend to avoid risk when perceiving higher risks, which diminishes their desire to re-participate. As mountaineering becomes increasingly popular among the general public, most participants lack professional skills and experience (Rebelo Miguel et al., 2023). During the activity, common risks such as adverse weather, complex terrain, and physical exhaustion heighten individual sensitivity to risk, thereby suppressing the likelihood of re-participation (Ahn and Song, 2024). Based on these insights, the following hypothesis is proposed:

*H1*: Risk perception negatively predicts re-participation intention.

### 2.2 Mediating effects of smooth experience

From the perspective of Flow Theory, different facets of perceived risk may undermine the conditions necessary for achieving a smooth experience. For instance, environmental risks (e.g., rapidly changing weather) can heighten vigilance and distract attention from task execution; equipment-related risks can diminish perceived control and disrupt clear action-feedback loops; and physiological risks (e.g., fatigue) can upset the balance between perceived challenges and personal skills, thereby increasing anxiety and reducing the likelihood of entering the flow state. A smooth experience arises when participants achieve a dynamic balance between their perceived skills and the challenges presented by the activity, leading to an optimal psychological state characterized by deep enjoyment and a sense of mastery (Kim et al., 2024). In this study, smooth experience is defined as a state where participants fully immerse their mental energy in mountaineering, becoming absorbed in the interaction with the external environment, with heightened interest, focused attention, and a sense of time loss, all accompanied by positive emotional experiences. Research in sports psychology shows a significant negative correlation between anxiety and smooth experience (Sotos-Ruiz et al., 2024). Specifically, when an individual’s subjective assessment of risk challenges exceeds their skill level and sense of control, anxiety increases, reducing the likelihood of entering a flow state, which in turn negatively impacts re-participation intention. From a psychological perspective, the relationship between risk perception and smooth experience is influenced by both cognitive evaluations and emotional responses. When participants perceive risks that exceed their coping abilities, they may experience anxiety, fear, and a sense of threat, which inhibits the occurrence of smooth experience. Thus, risk perception may serve as a barrier to achieving smooth experience.

Self-actualization Theory suggests that humans are inherently driven to pursue self-improvement, and smooth experiences are inherently positive psychological states (Chen et al., 2023). Once attained, they motivate repeated engagement. Therefore, it can be posited that smooth experience positively influence re-participation intention. The complexity of the mountaineering environment and the challenges it presents require individuals to focus more intensely on the activity itself. When participants experience high levels of immersion and a sense of control during the activity, their sensitivity to external potential threats may decrease, thus mitigating the inhibitory effect of risk perception on re-participation intention. Yang et al.’s (2025) study indicates that smooth experience can regulate individual anxiety levels, reduce excessive concern about risks, and enhance participants motivation to engage. Research also confirms that experience satisfaction positively affects individuals re-participate intention. Participants who experience positive outcomes are more satisfied, which facilitates the formation of positive re-participate intention (Cid et al., 2025). Smooth experience, as a form of positive emotion, significantly impacts both satisfaction and behavioral intentions (Ahn and Song, 2024). Bayram et al. (2025) found that participants who were satisfied with their leisure experience were more likely to recommend the destination to others, with experience quality having a significant positive effect on re-participation intention. In mountain sports, the more smooth experience participants gain from the activity, the fewer negative emotions they experience, and sustained smooth experience enhance their interest in the activity. This, in turn, improves the quality of the mountaineering experience and generates a cumulative positive emotional effect, which influences re-participation intention. Based on the above analysis, the study proposes the following hypotheses:

*H2*: Risk perception negatively predicts smooth experience.

*H3*: Smooth experience positively predicts re-participation intention.

*H4*: Smooth experience mediates the relationship between risk perception and re-participation intention.

### 2.3 Moderating effects of social support

Social support refers to the emotional, informational, and material assistance individuals receive from social relationships, such as family, friends, colleagues, or group organizations (Bayih and Singh, 2020). Social support provides emotional care and reduces the adverse effects caused by risk perception. Research indicates that parental, teacher, and peer support play significant positive moderating roles between exercise motivation and exercise persistence. Moreover, social support is closely related to exercise intention and environmental factors, exerting varying degrees of positive impact on physical activity participation (Wiltshire and Stevinson, 2018). Social support is a key factor in transforming participants’ cognitive and emotional states into effective decision-making behaviors. By adjusting their self-perception, individuals are able to release self-control in response to perceived risk, with social support providing the resources to help individuals better unleash their potential and enhance their re-participation intention. Social support alleviates psychological stress and reduces emotional tension, improving individuals’ adaptability to the environment (Zhang and Zhang, 2022).

**Figure 1:**
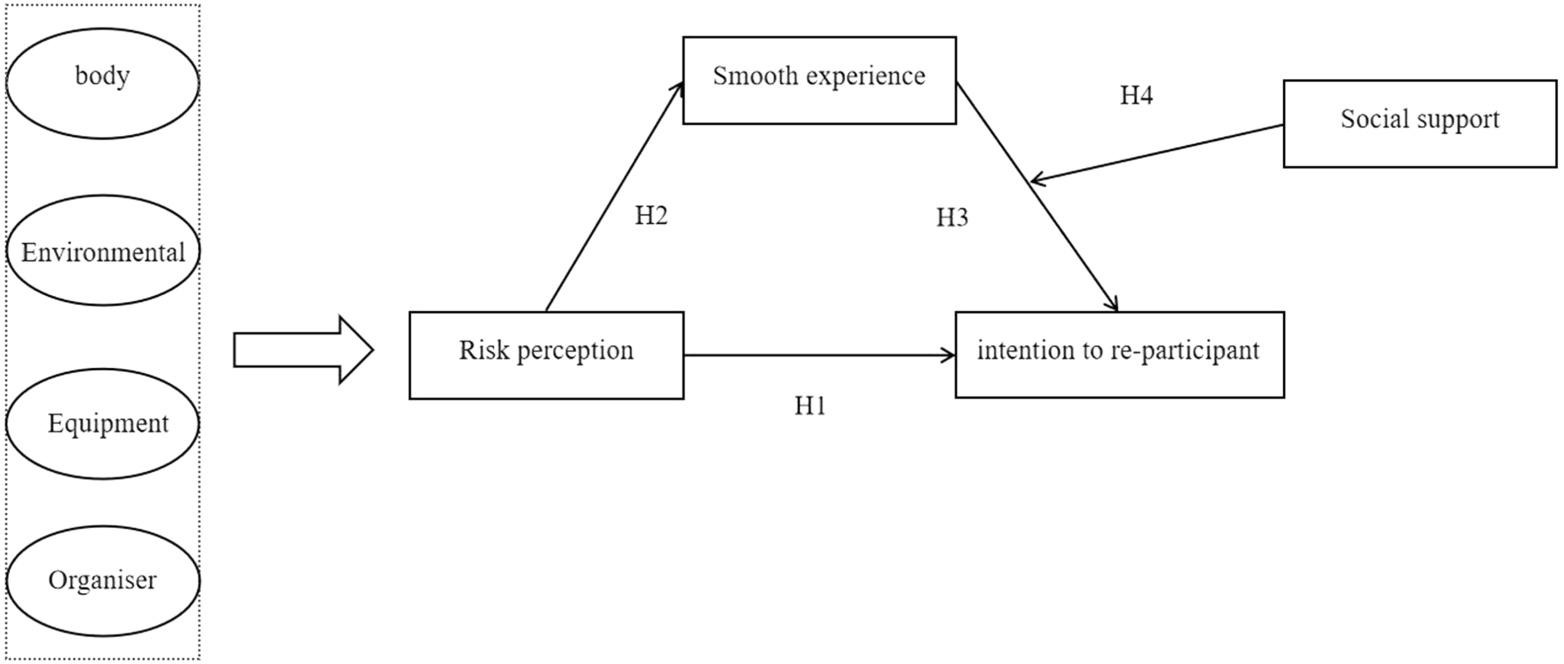
Proposed moderated mediation model.

Previous studies have shown that motivation-focused communication and positive atmospheres can foster positive emotional states by enhancing individuals’ psychological engagement and perceived support (Ntoumanis and Standage, 2009). Positive emotions, in turn, have been identified as a key psychological condition facilitating the emergence of smooth experience in sport and physical activity contexts (Rogatko, 2009; Harris et al., 2021). Through the generation of smooth experience, individuals deepen their intention to continue participating. In sports, high levels of social support provide participants with more information, emotional support, and material resources. This not only helps participants strengthen their decision-making but also boosts their confidence and self-identity, reducing the emotional distress caused by risk perception and minimizing the negative effects of anxiety. Furthermore, by enhancing positive emotional experiences, social support indirectly promotes re-participation intentions (Cohen and Wills, 1985). Thus, in mountaineering, strong team collaboration, peer encouragement, and coaching guidance enhance smooth experience, which in turn increases the intention to re-participate. Based on the above, the following hypothesis is proposed:

*H5*: Social support moderates the relationship between smooth experience and re-participation intention (Figure 1).

## 3 Methods

### 3.1 Participants

This study enrolled participants who had previously climbed outdoor peaks above 2,000 meters in altitude. The survey was conducted using convenience sampling from March to May 2025. Inclusion criteria for the sample were: (1) Age between 18 and 55 years; (2) Non-professional mountaineers or athletes; (3) At least one climb of a similar peak in the past year; (4) Ability to independently complete the questionnaire and voluntary participation in the study. Exclusion criteria included: (1) Professional mountaineers or related practitioners; (2) No climbs of similar peaks in the past year; (3) Incomplete questionnaire responses or obvious logical errors. Paper questionnaires were distributed on-site at major outdoor clubs or other easily accessible locations. Questionnaires were returned to participants after their climbs to assess their willingness to revisit similar peaks. Participants rated their willingness on a 1–5 scale. Sample selection adhered to the principles of “voluntary participation” and “immediate cooperation,” avoiding the screening of individuals with specific characteristics to ensure convenience and randomness in sample selection. To ensure the authenticity and completeness of questionnaire data, investigators explained the survey purpose and procedures to participants before completing the questionnaires. This allowed respondents to fully understand the research topic, reflect on their participation, and ensure timely collection and review of completed questionnaires. The study strictly complied with relevant regulations. After obtaining written informed consent, participants were required to complete paper questionnaires. Since the study did not involve minors, parental or guardian consent was not required. The study was conducted in three rounds, with 563 questionnaires distributed and 516 valid responses collected, yielding an effective response rate of 91.7%.

Analysis of the Valid Sample from the Official Survey: The gender distribution of the valid sample was relatively balanced, with males accounting for 45.3% and females 54.7%. The majority of respondents were young and middle-aged, with 43.1% aged 18–25 and 29.9% aged 26–35, while 5% were aged 50 and above. In terms of outdoor activity enjoyment, most participants enjoyed mountaineering, with 79% indicating they found the activity enjoyable. Regarding mountaineering experience and risk coping skills, a significant proportion of participants had limited experience, with 33.3% having participated for only 1 year and the majority having less than 3 years of experience. The primary motivations for participation were stress relief and scenic appreciation, with 72% of respondents citing these reasons.

### 3.2 Measures

The questionnaire comprises three sections: (1) As this study targets individuals with outdoor mountaineering experience, the questionnaire includes a screening question: ‘Do you have outdoor mountaineering experience?’ Respondents with experience proceed with the questionnaire; those without experience cease answering. (2) Personal information survey, including age, gender, years of sporting activity, coping skills, and enjoyment of adventure; (3) Core content measurement, covering latent variables such as risk perception, smooth experience, social support, and re-participation intentions. All variables are based on established scales and have been adapted to suit the characteristics of outdoor mountaineering. All items are scored using a five-point Likert scale.

Risk perception was measured using a scale developed by Bentley et al. (2007). This scale comprises six measurement items assessing risks related to the body, natural environment, project equipment, organizers, and other aspects. Responses were rated on a 5-point Likert scale (1 = Strongly Disagree, 5 = Strongly Agree). Higher average scores indicate stronger risk perception. Following exploratory and confirmatory factor analysis, the model fit indices were: *χ*^2^/df = 1.032, GFI = 0.994, RMSEA = 0.008, CFI = 1.000, NFI = 0.989, TLI = 0.999. Reliability testing revealed a Cronbach’s *α* coefficient of 0.816.

Smooth experience was measured using the scale revised by Yu and Chen (2021). This scale comprises five items primarily designed to assess sense of control, awareness merging into action, degree of concentration, and state of self-forgetfulness. Items were rated on a 5-point Likert scale (1 = Strongly Disagree, 5 = Strongly Agree). Higher average scores indicate stronger smooth experiences. Following exploratory and confirmatory factor analysis, the model fit indices were as follows: *χ*^2^/df = 1.903, GFI = 0.996, RMSEA = 0.000, CFI = 0.991, NFI = 0.979, TLI = 0.998. The reliability test results indicate that Cronbach’s *α* coefficient is 0.612.

Social support was measured using the Perceived Social Support Scale developed by Zimet et al. (1988). This scale comprises fifteen items assessing support received from parents, mentors, and peers during outdoor activities, rated on a 5-point Likert scale (1 = Strongly Disagree, 5 = Strongly Agree). Higher mean scores indicate stronger social support. Following exploratory and confirmatory factor analysis, the model fit indices were as follows: *χ*^2^/df = 1.938, GFI = 0.978, RMSEA = 0.001, CFI = 1.002, NFI = 0.976, TLI = 1.002. The reliability test results indicated a Cronbach’s *α* coefficient of 0.931.

The intention to re-participate was measured using Ajzen’s (1991) Theory of Planned Behavior and research by Kim et al. to assess participants’ willingness to re-participate (Wook, 2017). The scale comprised 4 items rated on a 5-point Likert scale (1 = Strongly Disagree, 5 = Strongly Agree). Higher mean scores indicate stronger intention to re-participate. Following exploratory and confirmatory factor analysis, the model fit indices were as follows: *χ*^2^/df = 1.283, GFI = 0.992, RMSEA = 0.002, CFI = 0.998, NFI = 0.998, TLI = 0.998. Reliability testing results indicate a Cronbach’s *α* coefficient of 0.929.

### 3.3 Data analysis

All analyses were performed using SPSS 27.0. To evaluate potential common method bias, Harman’s single-factor test was conducted. Additionally, confirmatory factor analysis (CFA) was performed to assess the measurement model and provide evidence for construct validity. Internal consistency was evaluated using Cronbach’s alpha coefficients.

The hypothesized moderated mediation model was tested using PROCESS macro (Model 14). This model tested whether smooth experience mediated the relationship between risk perception and re-participation intention, and whether social support moderated the path from smooth experience to re-participation intention (the second stage of the mediation). Gender, age, and mountaineering experience were included as control variables in all analyses to account for potential confounding effects and to more accurately estimate the relationships among the core psychological constructs. The bias-corrected percentile bootstrap method with 5,000 resamples was used to estimate indirect effects and generate 95% confidence intervals (CIs). Effects were considered statistically significant if the CIs did not include zero.

## 4 Results

### 4.1 Common method bias

In this study, the variables were assessed primarily through participants’ self-reported evaluations, which may introduce the risk of common method variance (CMV). To address potential CMV issues, both procedural and statistical controls were employed. Procedurally, participants were informed that the survey was anonymous and that the data would be used solely for academic purposes. In addition, the conceptual meaning of the study was explained to participants, and the order of questionnaire items was randomized to minimize response bias. Statistically, Harman’s single-factor test was used to detect common method bias. The results showed that two factors had eigenvalues greater than 1, and the first factor accounted for 37.51% of the variance, which is below the critical threshold of 40%. Therefore, CMV was not considered a significant issue in this study.

### 4.2 Variable correlation analysis

This study employed Pearson correlation analysis using SPSS 27.0 software to examine risk perception, smooth experience, social support, and intention to re-participate. Descriptive statistics revealed: risk perception exhibited significant negative correlations with both smooth experience and intention to re-participate (*p* < 0.01); social support and intention to re-participate showed a significant positive correlation (*p* < 0.01); smooth experience and intention to re-participate also exhibited a significant positive correlation (*p* < 0.01) (Table 1).

**Table 1:** Descriptive statistics and correlations among study variables (*N* = 516).

Variables	*M*	SD	1	2	3	4
Risk perception	3.769	0.594	1			
Smooth experience	1.494	0.39	−0.512^**^	1		
Social support	3.005	0.721	−0.034	0.02	1	
Re-participation intention	3.154	1.078	−0.410^**^	0.564^**^	0.659^**^	1

^*^p < 0.05, ^**^p < 0.01, ^***^p < 0.001.

### 4.3 Hierarchical regression

#### 4.3.1 Mediation effect analysis

The mediating effect of smooth experience in the relationship between risk perception and re-participation intention was examined using Model 14 of PROCESS macro (Version 4.1) for SPSS. The bootstrap sample size was set to 5,000, and the significance level of the confidence interval was set at 95%. A mediating effect is considered significant when the confidence interval does not include zero. Regression analyses were conducted for all variables in the research model. First, the total effect of risk perception on re-participation intention was tested, followed by examination of the significance of each path coefficient after including smooth experience as a mediating variable.

The results showed that risk perception significantly negatively predicted re-participation intention (*β* = −0.755, *t* = −10.138, *p* < 0.01), supporting Hypothesis H1. Risk perception also significantly negatively predicted smooth experience (*β* = −0.337, *t* = −13.274, *p* < 0.01), supporting Hypothesis H2 (Table 2).

**Table 2:** Regression results for the mediation model.

Variables	Re-participation intention (1)				Smooth experience (2)				Re-participation intention (3)			
	*B*	SE	*t*	*β*	*B*	SE	*t*	*β*	*B*	SE	*t*	*β*
Gender	0.132	0.089	1.478	0.061	0.004	0.031	0.136	0.005	0.127	0.08	1.585	0.059
Age	0.066	0.069	0.963	0.04	0.029	0.023	1.252	0.049	0.027	0.061	0.447	0.017
Mountaineering experience	0.009	0.013	0.749	0.032	0.004	0.004	1.049	0.043	0.003	0.011	0.31	0.012
Mountaineering skills	0.069	0.051	1.342	0.062	0.011	0.017	0.609	0.027	0.055	0.046	1.195	0.049
Enjoy mountaineering	−0.014	0.051	−0.283	−0.012	−0.018	0.017	−1.045	−0.043	0.009	0.045	0.209	0.008
Risk perception	−0.755**	0.074	−10.138	−0.415	−0.337**	0.025	−13.274	−0.513	−0.310**	0.078	−4.004	−0.171
Smooth experience									1.317**	0.118	11.163	0.477
*R*^2^	0.177				0.268				0.344			
Adj.*R*^2^	0.167				0.259				0.335			
ΔR^2^					0.091				0.167			
*F*	18.245^***^				31.059^***^				38.056^***^			

ΔR^2^ indicates the change in *R*^2^ after adding smooth experience; ^*^p < 0.05, ^**^p < 0.01, ^***^p < 0.001.

To further verify the mediating effect of smooth experience, the bootstrap method was employed (95% confidence interval, 5,000 resamples). The results indicated that the indirect effect of the pathway “risk perception → smooth experience → re-participation intention” was −0.444, with a 95% confidence interval of [−0.295, −0.196], which did not include zero. This confirms the existence of a significant mediating effect. This result confirms that smooth experience significantly mediates the relationship between risk perception and re-participation intention, supporting Hypotheses H3 and H4 (Table 3).

**Table 3:** Decomposition of total, direct, and mediating effects.

Effect	Path	*B*	SE	LLCI	ULCI
Total effect		−0.755	0.074	−0.901	−0.608
Direct effect	Risk perception →Re-participation intention	−0.310	0.078	−0.463	−0.158
Indirect effect	Risk perception → Smooth experience → Re-participation intention	−0.444	0.025	−0.493	−0.395

Indirect effects were estimated using 5,000 bootstrap resamples. Bootstrap sample size = 516, LL, Lower level; UL, Upper level.

#### 4.3.2 Moderation effect analysis

Social support serves as an important external resource that influences individual motivation and behavioral intentions in mountaineering contexts. To examine its moderating role, we tested whether social support moderates the relationship between smooth experience and re-participation intention, corresponding to the second stage of the mediation model. The results showed that the interaction term between smooth experience and social support significantly predicted re-participation intention, indicating that social support strengthens the positive association between smooth experience and re-participation intention. Therefore, Hypothesis H5 was supported (Table 4).

**Table 4:** Moderating effect of social support on the relationship between smooth experience and re-participation intention.

Variables	Re-participation intention			Smooth experience		
	*β*	SE	*t*	*β*	SE	*t*
Gender	0.002	0.049	0.048	0.004	0.031	0.136
Age	0.002	0.037	0.041	0.029	0.023	1.252
Mountaineering experience	0.003	0.007	0.493	0.004	0.004	1.049
Mountaineering skills	0.003	0.028	0.107	0.011	0.017	0.609
Enjoy mountaineering	0.036	0.028	1.303	−0.018	0.017	−1.045
Risk perception	−0.266^**^	0.047	−5.623	−0.337	0.025	−13.274
Social support	0.579^**^	0.136	4.245			
Smooth experience	0.557^*^	0.272	2.048			
Smooth experience × Social support	0.253^**^	0.087	2.908			
*R*^2^	0.541			0.268		
Adj. R^2^	0.533			0.259		
Δ*R*^2^	0.273					
*F*	66.250^***^			31.059^***^		

*β* indicates standardized regression coefficients. Δ*R*^2^ represents the increase in explained variance after adding the interaction term (Smooth experience × Social support). ^*^p < 0.05, ^**^p < 0.01, ^***^p < 0.001.

**Figure 2:**
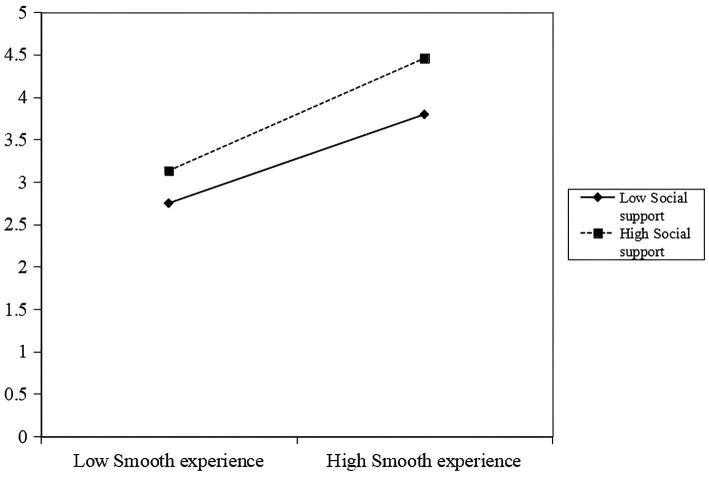
Simple slope plot of the moderating effect of social support on the relationship between smooth experience and re-participation intention.

To further examine the interaction between smooth experience and social support, a simple slope analysis was conducted (Figure 2). Social support was categorized into high and low levels (M ± 1 SD), and an interaction plot was generated to illustrate the relationship between smooth experience and re-participation intention across different levels of social support. The results showed that smooth experience was positively associated with re-participation intention under both high and low levels of social support. However, the strength of this association was greater for individuals reporting higher levels of social support. These findings indicate that as social support increases, the positive effect of smooth experience on re-participation intention becomes stronger, thereby supporting the moderating role of social support in this relationship.

## 5 Discussion

### 5.1 Mechanism between risk perception and re-participation intention

This study demonstrates that risk perception exerts a significant and direct negative effect on individuals’ intention to re-engage in mountaineering. Specifically, stronger perceptions of mountaineering-related risk are associated with more negative overall evaluations of the activity and a more pronounced tendency toward behavioral avoidance. This finding is consistent with a substantial body of prior research identifying risk perception as a key psychological barrier to participation. It further corroborates the view that risk aversion remains a dominant factor constraining sustained engagement in mountaineering and other high-risk outdoor pursuits (Li et al., 2023). However, necessary to note that not all studies view risk perception as a purely negative factor. Studies examining high-risk and extreme sports contexts reveal that individuals possessing strong sensation-seeking or risk-preference traits often do not retreat in the face of heightened risk perception. Instead, they actively pursue risk to attain experiences of excitement, challenge, and self-transcendence (Apollo et al., 2023). From this perspective, perceived risk can function as an “attractive stimulus,” enhancing participation motivation and engagement intensity. These seemingly divergent findings suggest that the behavioral effects of risk perception are not universal but are jointly shaped by individual characteristics and contextual conditions (Jackman et al., 2023). A key reason for these discrepancies lies in participant typology. Studies emphasising risk attractiveness predominantly involve experienced athletes, professional adventurers, or individuals with high sensation-seeking traits. For such populations, risk is often assessed as controllable and intrinsically rewarding. In contrast, the present study primarily examines non-professional mountaineers, a significant proportion of whom lack systematic training and extensive experience. For this group, risks are more readily perceived as unpredictable and threatening, triggering anxiety and avoidance behaviors rather than excitement and challenge motivation. This discrepancy indicates that risk perception may serve distinctly different motivational functions across participant types.

Compared with mountaineering, risks encountered in traditional sports—such as ball games, fitness activities, or conventional competitive events—are generally more predictable and controllable. These risks typically arise from technical errors, physical exertion, or interpersonal contact and occur within environments governed by clear rules, familiar venues, and well-established safety protocols. In such contexts, risk is more readily perceived as an integral and manageable component of the sporting process and may even be interpreted as a challenging stimulus that enhances participation motivation, even interpreted as challenging stimuli, thereby exerting a motivational effect on participation. In contrast, the risk profile inherent in mountaineering exhibits markedly different structural characteristics. Mountaineering risks derive not only from individual skill level or physical fitness but also from complex and highly uncertain external factors, including dynamic natural environmental conditions, altitude-induced physiological stress, equipment reliability, and the adequacy of organizational and management safety systems. Many of these risks exceed an individual’s immediate capacity for control. When such risks are perceived as externally imposed and unpredictable threats, participants’ psychological responses are more likely to manifest as anxiety and heightened vigilance rather than challenge-oriented motivation. Consequently, the negative association between risk perception and re-participation intention observed in this study reflects the tendency for mountaineering risks to be interpreted as threatening constraints rather than motivating challenges, thereby diminishing positive experiential outcomes and inhibiting sustained participation willingness.

Within China’s recreational sports sector, mountaineering attracts growing numbers of participants alongside expanding demand for adventure-based leisure activities and outdoor pursuits. However, this sport demands not only sound physical fitness but also fundamental safety awareness (Yu et al., 2024). Due to insufficient training systems and incomplete regulatory frameworks, many novice participants tend to overestimate potential hazards, which in turn reduces their willingness to participate (Nam and Yang, 2025). Prior research indicates that higher perceived destination risks correlate with lower participation likelihood. For instance, individuals entering high-altitude or technically challenging environments without systematic preparation often experience excessive anxiety, diminishing overall experience quality and weakening participation intent (Zeng et al., 2024). Existing research further indicates that mountain outdoor enthusiasts value natural environments, experiential quality, and social security systems. Strong risk perceptions frequently impede the formation of positive participation intent. Consequently, outdoor activity managers typically regard risk mitigation as a key strategy for enhancing participation levels.

Overall, the role of risk is not universally applicable but is jointly shaped by participant type, risk-taking characteristics, and socio-cultural context. Overall, risk perception constitutes a key psychological barrier affecting sustained participation in mountaineering. As mountain outdoor recreation continues to evolve, refining public safety systems, reducing perceived risk, and optimizing the mountaineering experience have become central pathways to meeting participants’ psychological needs and stimulating positive behavioral intention. Consequently, enhancing risk management capabilities and strengthening safety education represent fundamental prerequisites for lowering risk perception and increasing participation rates.

### 5.2 Mechanism of smooth experience between risk perception and re-participation intention

Smooth experience not only directly affects re-participation intention but also serves as a key mediating mechanism through which risk perception influences subsequent behavioral decisions. This study demonstrates that risk perception alters individuals’ attention focus, sense of control, and emotional states, thereby shaping the intensity and quality of smooth experience, which in turn impacts re-participation intention.

Measurement limitations should be acknowledged when interpreting the present findings. In particular, the internal consistency of the smooth experience scale was relatively low (Cronbach’s *α* = 0.612), falling below the commonly recommended threshold of 0.70. Although the scale demonstrated acceptable factorial validity and was adapted from previously validated instruments, the modest reliability indicates that smooth experience may not have been captured with optimal precision in the context of recreational mountaineering. From a methodological perspective, lower internal consistency may attenuate observed relationships by introducing measurement error, leading to conservative estimates of regression coefficients and indirect effects. Accordingly, the mediating role of smooth experience should be interpreted with caution, as the strength of the mediation pathway may be underestimated rather than inflated. Notably, the persistence of significant mediation effects despite limited reliability suggests that the underlying relationships are likely robust.

During mountaineering, when participants invest their full cognitive and emotional resources, they experience focused attention, deep involvement, and positive affect, which significantly strengthen participation intentions. As a core psychological mechanism for sustaining engagement and enhancing satisfaction, flow encompasses both cognitive and behavioral dimensions: cognitive flow includes the integration of self-awareness and action, heightened control, and temporal distortion, whereas behavioral flow is reflected in smooth task execution, instant feedback, and focused attentional engagement (Lise, 2023). Flow Theory posits that psychological states during activity are co-determined by external environmental conditions and internal cognitive evaluations. When perceived challenge matches individual skill level, flow is most likely to occur. Conversely, if perceived risk exceeds personal control capacity, anxiety increases and cognitive resources shift toward threat avoidance, inhibiting flow formation and reducing its intensity.

This study further finds that the negative effect of risk perception on re-participation intention is not irreversible. Under moderate risk conditions, if individuals receive positive emotional feedback and a sense of accomplishment, their re-participation intention may still increase. Thus, flow serves as a “psychological buffer” that mitigates the adverse impact of risk perception. This aligns with Rogatko’s (2009) conclusion that flow enhances positive emotional memory, offsets anxiety and fear, and promotes re-participation intention. In mountaineering, the interplay between risk perception, smooth experience, and re-participation intention is particularly complex (Boudreau et al., 2020). Excessive perceived risk diverts attention from the task to potential threats, triggers anxiety and fear, and disrupts immersion, thereby impairing flow formation. In contrast, moderate levels of risk may enhance alertness, trigger motivation, and foster deeper engagement. When individuals successfully enter a flow state and perceive strong control and competence, they experience “mind–body unity,” further reinforcing re-participation intention. According to Emotion Memory Effect, positive affect strengthens memory encoding and retrieval (Ayçiçeği-Dinn and Caldwell-Harris, 2009). Thus, even when participants initially perceive high risk, achieving flow can result in predominantly positive emotional memory, leading to greater future participation willingness. Practically, enhancing public re-participation intention requires: (1) reducing risk perception through safety education and psychological guidance; and (2) creating supportive conditions for smooth experience, such as optimizing route design, providing immediate feedback, and offering positive reinforcement to help participants maintain sustained engagement under manageable challenges. Only by advancing risk management and experiential optimization simultaneously can mountaineering participants maintain positive emotions, develop flow states, and form strong re-participation intention.

### 5.3 Mechanism of social support between smooth experience and re-participation intention

This study found that social support significantly moderated the relationship between smooth experiences and intention to participate again in mountaineering. Social support does not merely function by directly enhancing behavioral intentions; rather, it substantially influences how individuals translate positive experiential states into sustained participation intentions. To fully comprehend this moderating effect, it is necessary to delve into the underlying socio-psychological mechanisms within the context of high-risk outdoor activities.

From social psychological perspective, enhancing self-efficacy through social support constitutes a key mechanism. First, within the uncertain and physically demanding context of mountaineering, individuals gain crucial reference points by observing peers or teammates successfully complete similar tasks, thereby strengthening confidence in their own capabilities. This positive social support enhances individuals’ sense of competence and reduces uncertainty, facilitating the conversion of smooth experiences into motivation for repeated mountaineering participation. Second, the sharing of responsibilities among team members reduces subjective risk burdens. When mountaineering occurs within a supportive group context, decision-making, risk assessment, and task execution are often collectively undertaken by team members. This collective involvement helps diffuse individual responsibility for potential hazards, thereby reducing perceived subjective risk burden (Liu et al., 2024). Consequently, individuals are less likely to interrupt smooth experiences due to excessive risk anxiety, allowing positive emotional and attention states to persist and further translate into future re-participation intention. Thirdly, social support performs crucial emotional regulation functions within high-stress contexts. According to the buffering effect model of social support, both affective and instrumental support effectively alleviate fear, anxiety, and tension arising during mountaineering activities (Wang and Wang, 2025). A supportive social atmosphere enhances psychological safety and attention focus, enabling participants to maintain an immersive state even in the case of uncertain environmental risks. This process aligns with Social Identification Theory, which posits that strong group belonging enhances individual emotional stability and behavioral consistency (Saripanidis et al., 2025). Within cohesive mountaineering teams, shared objectives and collective identity prolong the duration of smooth experiences and amplify their motivational impact on behavioral intention. From the resource conservation perspective, social support constitutes a vital external resource, aiding individuals in countering resource depletion during high-risk scenarios (Hagger, 2015). Mountaineering demands sustained physical and mental investment, rendering participants vulnerable to emotional exhaustion and diminished motivation. Social support mitigates resource depletion by providing affective, informational, and instrumental resources, thereby sustaining psychological energy levels. This enables smooth experiences to exert a more enduring influence on re-participation intentions even within highly uncertain and potentially hazardous environments (Wei et al., 2025).

Unlike conventional sports, mountaineering relies more heavily on social resources to shape individuals’ cognitive support regarding risk and responsibility. Social support not only amplifies the motivational effects of smooth experiences but also facilitates the conversion of optimal experiences into sustained participation by reshaping risk perception and responsibility allocation. Practically, strengthening social support systems is vital for the sustainable development of mountaineering. On one hand, professional mountaineering services, medical support, and information provision enhance participants’ sense of security and experience quality; on the other hand, cultivating supportive community environments through social media platforms, interest groups, and volunteer networks facilitates communication and mutual aid among participants, fostering an atmosphere of ‘collective smooth experiences.’ Thus, social support not only acts as a buffer but also serves as a key facilitator in promoting social connection, enhancing belonging and trust, sustaining positive emotions, and prolonging smooth experiences, ultimately increasing the intention to revisit mountaineering activities.

## 6 Conclusion and recommendations

### 6.1 Conclusion

Building on Risk Perception Theory and Flow Theory, this study develops and empirically tests a behavioral model to explain individuals’ re-participation intention in mountaineering. The findings indicate that risk perception, smooth experience, and social support jointly determine re-participation intention.

First, risk perception functions as a core inhibitory factor: higher levels of perceived risk significantly diminish re-participation intention. Second, risk perception not only exerts a direct negative effect on re-participation intention but also indirectly influences it via the mediating role of smooth experience. Finally, social support plays a significant positive moderating role in the latter stage of the mediating process; specifically, at higher levels of social support, the positive effect of smooth experience on re-participation intention is strengthened.

### 6.2 Recommendations

#### 6.2.1 Organizational strategies: enhancing smooth experiences and social support systems

Mountaineering organizers, clubs, and instructors play a critical role in shaping participants’ psychological and experiential outcomes. First, scientific route planning and activity stratification are needed. Establish multi-level routes tailored to varied physical and technical abilities, ensuring an appropriate challenge–skill balance conducive to flow states. Second, design structured engagement processes by pacing activities reasonably, incorporating instructional and interactive sessions, and offering timely encouragement and technical guidance to improve immersion and confidence. Third, deploy professional coaching and safety teams, provide standardized safety protocols, real-time feedback, and psychological reassurance, effectively reducing perceived risks associated with uncertainty and inexperience. These measures collectively improve participation quality, create supportive environments, and enhance long-term engagement.

#### 6.2.2 Individual strategies: strengthening self-regulation and risk response capacity

Mountaineering participants should actively cultivate scientific risk cognition, enhance emotional resilience, and develop technical competence to better cope with environmental uncertainty. First, prioritize physical and technical preparation by engaging in systematic training, improving endurance and strength, and mastering essential mountaineering skills to ensure alignment between personal capability and activity difficulty. Second, adopt rational risk appraisal and psychological adjustment strategies. Approach perceived risks as manageable rather than prohibitive, practice anxiety-management techniques, such as breathing exercises and mindfulness, and maintain a positive mindset throughout the climbing process. Through these strategies, individuals can strengthen their self-efficacy, enter flow states more readily, and develop sustained motivation for continued participation.

## 7 Research limitations and prospects

Despite yielding meaningful theoretical and practical insights, this study has several limitations that provide avenues for future research. First, it must be acknowledged that the representative sample has limitations. Due to practical constraints, convenience sampling was adopted, and although participants had all climbed peaks above 2000 meters, the sample was primarily drawn from specific regions and outdoor communities. This sampling approach may have resulted in a relatively homogeneous sample and introduced potential selection bias, thereby limiting the external validity and universality of the findings to broader populations, such as novice hikers, professional climbers, or individuals engaged in other types of mountain activities. Future research is encouraged to adopt more rigorous sampling strategies, such as random or stratified sampling across diverse geographic regions, experience levels, and social backgrounds, to enhance sample universality and external validity.

Second, The reliability of measuring smooth experience. The relatively low internal consistency of this scale may have weakened the observed correlations with smooth experience. Although structural validity is supported, future research should refine the operational definition of smooth experience in mountaineering contexts by adopting more context-sensitive measurement tools or revising measurement models.

## Data Availability

The raw data supporting the conclusions of this article will be made available by the authors, without undue reservation.
